# Perforation of the Knee Joint Following Antegrade Intramedullary Nailing of a Comminuted Femoral Diaphyseal Fracture: A Case Report

**DOI:** 10.7759/cureus.24747

**Published:** 2022-05-05

**Authors:** Michael G Flood, Benjamin J Wie, Matthew P Sullivan

**Affiliations:** 1 Department of Orthopedic Surgery, State University of New York (SUNY) Upstate Medical University, Syracuse, USA

**Keywords:** knee injuries, effusion, antegrade intramedullary nail, femoral shaft fractures, interlocking nailing, comminuted fracture

## Abstract

A 63-year-old man with a comminuted spiral femoral shaft fracture was treated with closed reduction and internal fixation with a cephalomedullary nail. Two weeks postoperatively, one of the two static distal interlocking bolts began backing out and was removed. The nail ultimately migrated distally and perforated the knee joint at four months postoperatively. The patient was successfully treated with an exchange nail and percutaneous bone graft to the fracture site.

A single static distal interlocking bolt may be inadequate to maintain length in a healing comminuted spiral femur shaft. Multiple distal interlocking bolts should be in place until the completion of fracture healing.

## Introduction

This case represents a unique complication of intramedullary nailing (IMN) of a femoral fracture. While many studies have explored the incidence of anterior cortex penetration by the femoral nail intraoperatively [1-4], few have documented this occurrence in the postoperative period. In fact, only one report has described such a situation. Fantry et al. describe a patient who suffered nonunion after placement of a trochanteric entry recon nail. He underwent nail dynamization with the removal of the distal interlocking screw to allow compression through the nonunion. The patient was then lost to follow up for three years and returned for knee pain and stiffness and was found to have persistent nonunion with nail perforation into the knee. Ultimately, it was decided to remove the intramedullary nail and perform revision nailing with dynamic compression [5]. There are no reports, however, of patients with nonunion and intramedullary nail perforation of the knee joint following removal of the distal interlocking bolt without dynamization.

## Case presentation

**Figure 1:**
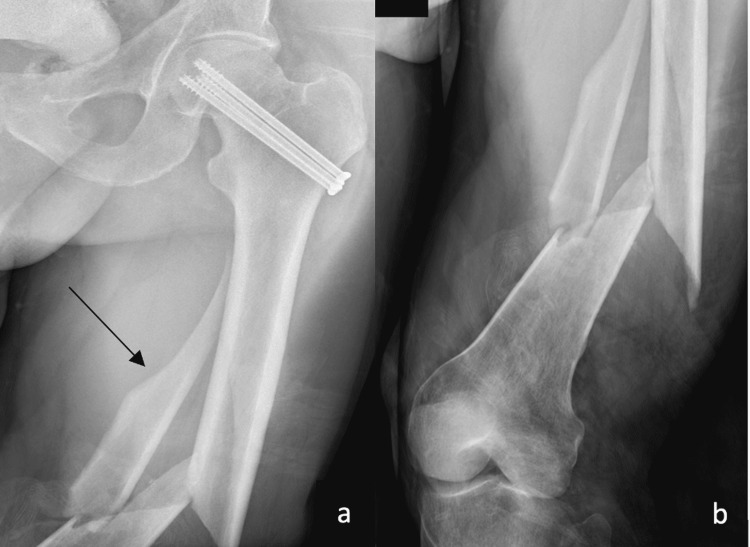
X-rays of the injury x-rays demonstrating spiral, comminuted diaphyseal femoral fracture. a) AP view of the proximal femur, with cannulated screws from a prior surgery in the femoral neck, and arrow to one of the fracture fragments; b) AP view of the distal femur showing the comminuted fracture AP: anteroposterior

A 63-year-old male sustained a closed left femoral shaft fracture, AO type B1, in an all-terrain vehicle (ATV) accident (Figure 1). The patient's medical history was significant for osteopenia, treated with testosterone replacement therapy. He was later diagnosed with prostate cancer without skeletal metastases on a bone scan. Past surgical history was notable for an ipsilateral femoral neck fracture 13 years prior, which was treated with cannulated screws. The patient had no prior injuries or surgery to the contralateral femur.

**Figure 2:**
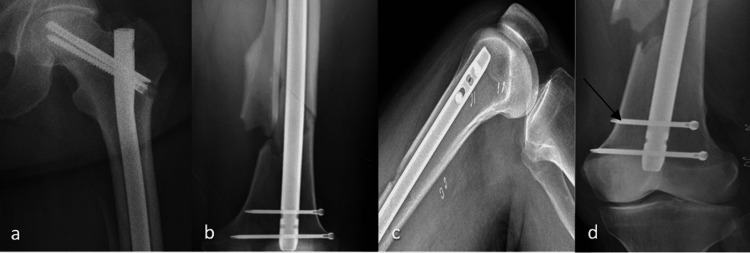
Postoperative imaging showing the intramedullary nail a) AP X-ray of the proximal femur with replacement of screws in femoral head; b) AP view of the distal femur showing the nail and two interlocking bolts; c) lateral view of the distal femur; d) AP view of the distal femur with an arrow to one of the two interlocking bolts AP: anteroposterior

**Figure 3:**
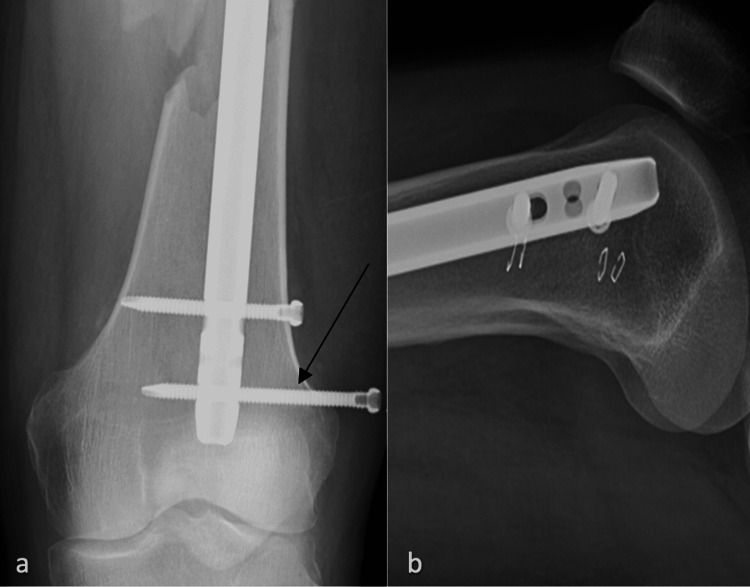
X-rays two weeks after the index operation a) AP view of the distal femur with the arrow to the more distal interlocking bolt backing out of the femur; b) lateral view of the distal femur AP: anteroposterior

**Figure 4:**
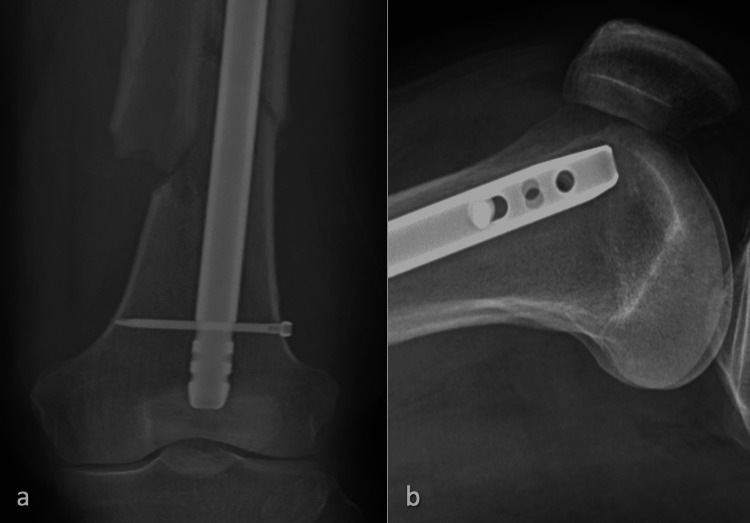
X-rays nine weeks after the index operation a) AP view of the distal femur; b) lateral view of the distal femur AP: anteroposterior

The patient was taken to the operating room on the day following the injury for closed reduction and cephalomedullary nail fixation of the femur fracture in conjunction with the removal of hardware from the left femoral neck. A Smith and Nephew TriGen Meta-Tan antegrade femoral nail size 13 mm x440 mm (London, United Kingdom) was used with two statically placed distal interlock bolts (Figure 2). Of note, the patient’s left femoral neck was slightly retroverted due to his prior femoral neck fracture in addition to his native retroversion (evaluated on XR of the contralateral hip). Femur shaft rotation was set to match the contralateral femur. Postoperatively, the patient was made weight-bearing as tolerated and did well until his two-week postoperative visit, at which time, the inferior distal interlocking bolt was noted to be backing out and tenting skin (Figure 3). The patient returned to the OR for the removal of this bolt. Intraoperatively, the remaining distal interlocking bolt was noted to be bicortical with no signs of failure. The patient remained on the weight of his leg flat foot for six weeks postoperatively in order to minimize stress across the single distal interlocking bolt, given the length unstable nature of his original fracture. The patient’s pain improved gradually, and he was able to mobilize using a walker. At nine weeks following the index procedure, XR demonstrated some distal migration of the nail (Figure 4). However, the patient was reporting continued symptomatic improvement, so the plan was to continue monitoring.

**Figure 5:**
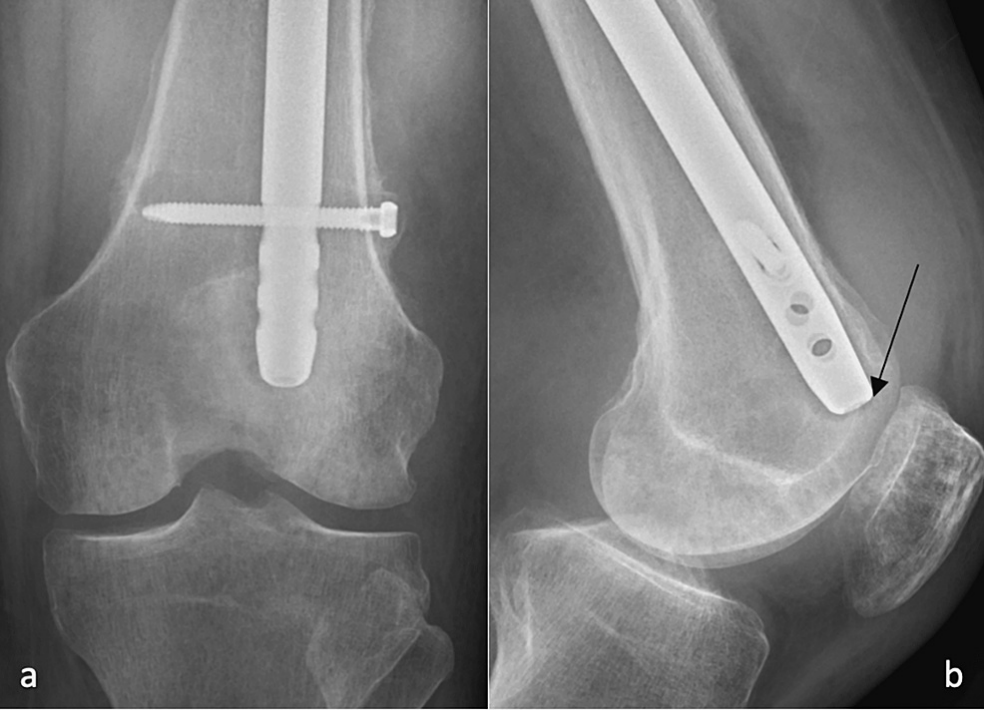
X-rays four months after the index operation a) AP view of the distal femur; b) lateral view of the distal femur with an arrow to the distal portion beginning to perforate the knee joint AP: anteroposterior

**Figure 6:**
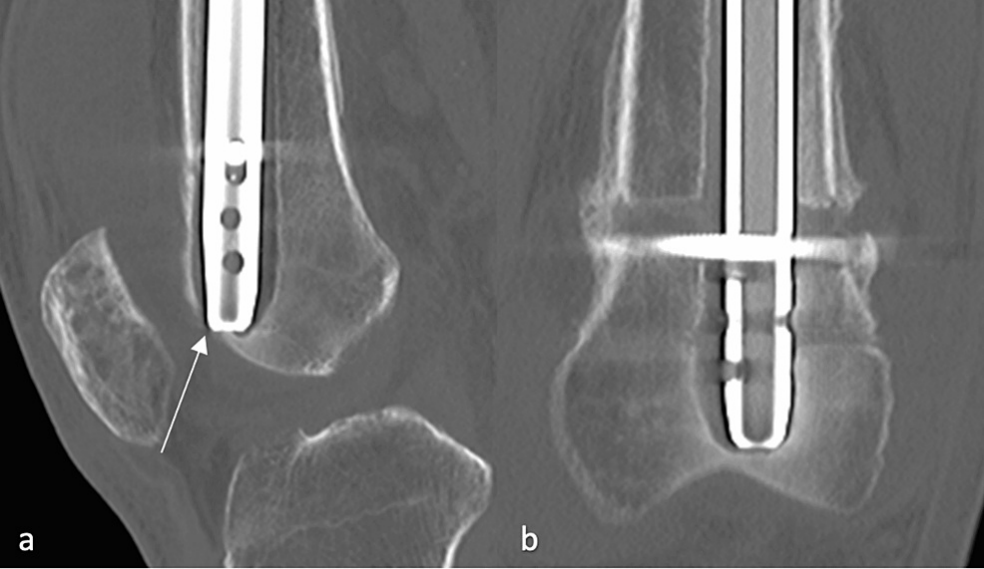
CT scan four months after the index operation a) Lateral view of the distal femur with the arrow to the distal nail penetrating the synovium of the knee; b) AP view of the distal femur AP: anteroposterior

**Figure 7:**
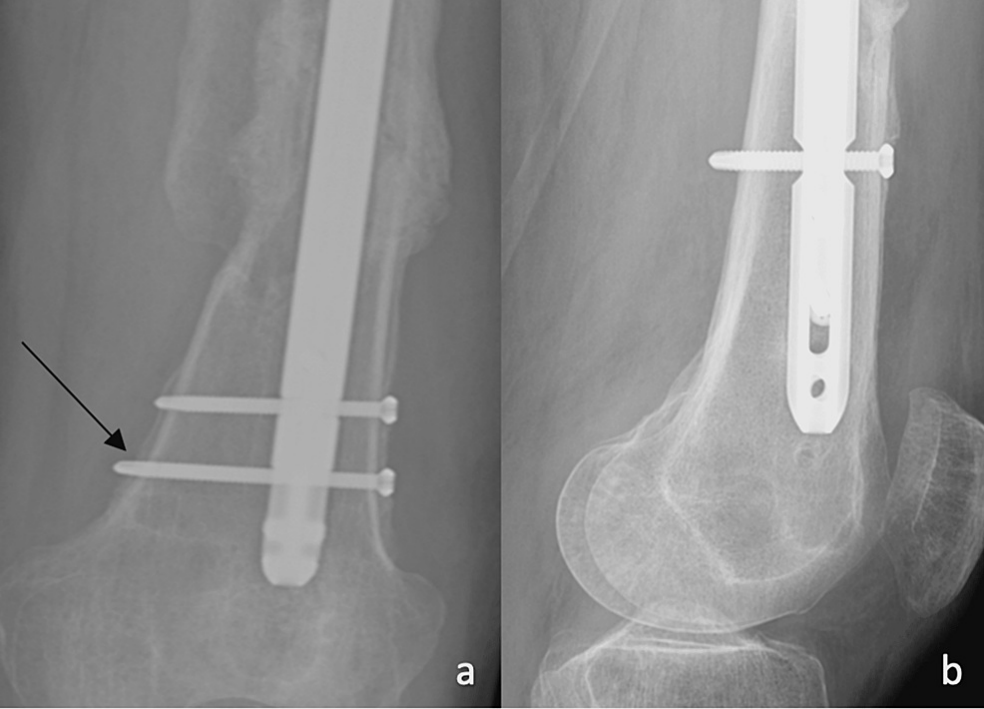
X-rays following the revision procedure a) AP view of the distal femur with the exchanged nail, with an arrow to one of the two replacement bolts; b) lateral view of the distal femur AP: anteroposterior

**Figure 8:**
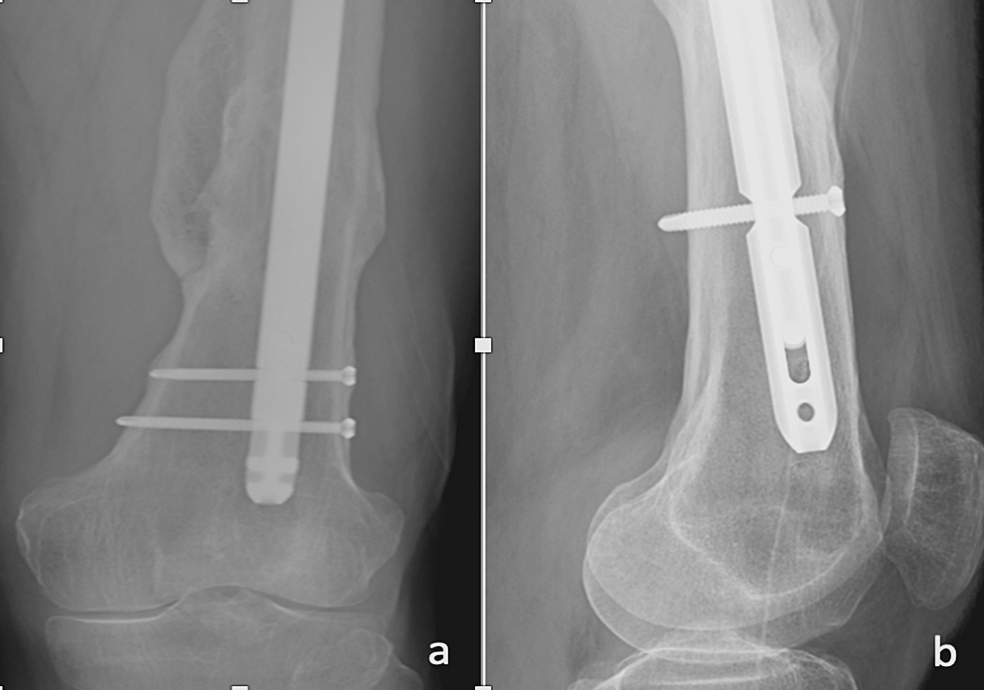
X-rays 1.5 years following the revision procedure a) AP view of the distal femur; b) lateral view of the distal femur AP: anteroposterior

At his four-month postoperative visit, the patient presented with a new left knee effusion and pain. X-rays at this time showed delayed union of the femoral shaft fracture, and the intramedullary nail had further migrated distally and anteriorly (Figure 5). Given the level of knee swelling, an infectious workup was initiated. Knee aspiration was significant for a total nucleated cell count of 71,800 but there was no growth on cultures. Due to persistent pain, the patient presented to the ED four days later where CT revealed the distal tip of the nail had perforated the anterior trochlea into the joint (Figure 6). His serum erythrocyte sedimentation rate (ESR)/C-reactive protein (CRP) was elevated, a repeat knee arthrocentesis returned a total nucleated cell count of 21,000, and once again, there was no growth on cultures. The patient was taken to the operating room (OR) for exchange nailing with nonunion repair with iliac autograft along with irrigation and drain placement in the knee. The original IMN was replaced with a Synthes femoral reconstruction nail (14 mm x 400 mm; DePuy Synthes Companies, Raynham, MA) for its smaller radius of curvature to decrease the risk of anterior perforation (Figure 7). Intraoperative cultures of marrow and knee aspiration were obtained and, ultimately, were negative for infection. The patient demonstrated marked improvement in mobility and pain over the acute postoperative period. At his most recent follow-up at 1.5 years after revision, the patient demonstrated complete bony union of the fracture with the resolution of knee pain and swelling (Figure 8).

## Discussion

Femoral shaft fractures are high-energy injuries, frequently associated with major trauma and additional injuries to other body systems, as well as ipsilateral femoral neck fractures. The typical management for this injury is an intramedullary nail. There is a strong biomechanical basis for the efficacy of an IMN. Cheung et al. explain that the nail “acts as an internal splint with load sharing characteristics,” which offer varying degrees of load-bearing through hardware depending on the fracture pattern. Comminuted and long oblique fractures rely more heavily on interlocking screws for stability due to their length unstable nature compared to transverse or short oblique fractures [6]. Karadimas et al. reported on 415 patients with femoral shaft fractures treated with intramedullary nailing, of which 179 were classified as AO type B [7]. Ten of these type B femur fractures went on to delayed union (defined as the absence of union after three months) and were either treated with nail dynamization or went on to union without intervention. There are various risk factors that increase the risk for nonunion, including obesity, hypertension, diabetes, fracture site at the proximal third of the femur, or a comminuted fracture pattern [8-9]. In addition to nonunion, another potential complication is the penetration of the femoral anterior cortex intraoperatively; several studies have explored the risk factors and prevention of this issue [1-4]. There are few cases in the literature of perforation of an intramedullary femoral nail into the knee joint.

This case report offers a unique complication in that the point of failure was the patient’s distal femur. When a loose distal interlocking bolt was removed two weeks following the patient’s index procedure, the single remaining static-locking distal bolt was not revised. Dynamization was not attempted due to the length-unstable fracture pattern.

In our evaluation, critical factors that contributed to the nonunion and perforation of the knee joint were fracture pattern and removal of one of the two distal interlocking bolts 2.5 weeks after the injury and index surgery. We hypothesize that the patient’s distal femur bone stock could not support load-bearing from the single interlocking bolt without replacement of the second, previously removed, screw, and allowed the fracture to shorten. Other ancillary factors may have contributed to this patient’s complication. A shorter nail length may have positioned the distal interlocking bolt closer to the diaphyseal bone and would have required significantly more shortening through the fracture before perforating the knee joint.

## Conclusions

This patient’s presentation is a rare complication of intramedullary nailing of femoral shaft fractures. The key observation is the failure of osteoporotic bone in the context of a single distal interlocking bolt. Therefore, we propose that a minimum of two interlocking bolts with a good bicortical purchase be maintained at all times to ensure adequate load balancing across the fixation until union is achieved, particularly in patients with poor bone stock. We believe this will be fundamental in preventing a similar complication from occurring in the future.
